# Loss of Integrin αvβ8 in Murine Hepatocytes Accelerates Liver Regeneration

**DOI:** 10.1016/j.ajpath.2018.10.007

**Published:** 2019-02

**Authors:** Stephen N. Greenhalgh, Kylie P. Matchett, Richard S. Taylor, Katherine Huang, John T. Li, Koy Saeteurn, Mhairi C. Donnelly, Eilidh E.M. Simpson, Joshua L. Pollack, Amha Atakilit, Kenneth J. Simpson, Jacquelyn J. Maher, John P. Iredale, Dean Sheppard, Neil C. Henderson

**Affiliations:** ∗ Centre for Inflammation Research, The Queen's Medical Research Institute, University of Edinburgh, Edinburgh, United Kingdom; † Lung Biology Center, Department of Medicine, University of California, San Francisco, San Francisco, California; ¶ Liver Center, Department of Medicine, University of California, San Francisco, San Francisco, California; § Department of Pathology, University of California, San Francisco, San Francisco, California; ‡ Department of Hepatology, Scottish Liver Transplant Unit and University of Edinburgh, Royal Infirmary of Edinburgh, Edinburgh, United Kingdom; ‖ Senate House, University of Bristol, Bristol, United Kingdom

## Abstract

Recent fate-mapping studies in mice have provided substantial evidence that mature adult hepatocytes are a major source of new hepatocytes after liver injury. In other systems, integrin αvβ8 has a major role in activating transforming growth factor (TGF)-β, a potent inhibitor of hepatocyte proliferation. We hypothesized that depletion of hepatocyte integrin αvβ8 would increase hepatocyte proliferation and accelerate liver regeneration after injury. Using *Itgb8*^*flox/flox*^*;Alb-Cre* mice to deplete hepatocyte αvβ8, after partial hepatectomy, hepatocyte proliferation and liver-to-body weight ratio were significantly increased in *Itgb8*^*flox/flox*^*;Alb-Cre* mice compared with control mice. Antibody-mediated blockade of hepatocyte αvβ8 *in vitro*, with assessment of TGF-β signaling pathways by real-time quantitative PCR array, supported the hypothesis that integrin αvβ8 inhibition alters hepatocyte TGF-β signaling toward a pro-regenerative phenotype. A diethylnitrosamine-induced model of hepatocellular carcinoma, used to examine the possibility that this pro-proliferative phenotype might be oncogenic, revealed no difference in either tumor number or size between *Itgb8*^*flox/flox*^*;Alb-Cre* and control mice. Immunohistochemistry for integrin αvβ8 in healthy and injured human liver demonstrated that human hepatocytes express integrin αvβ8. Depletion of hepatocyte integrin αvβ8 results in increased hepatocyte proliferation and accelerated liver regeneration after partial hepatectomy in mice. These data demonstrate that targeting integrin αvβ8 may represent a promising therapeutic strategy to drive liver regeneration in patients with a broad range of liver diseases.

Although the liver has a unique ability to regenerate, in many cases of liver disease this regenerative capacity is overwhelmed. A successful pro-regenerative therapy for the liver could have widespread application, reducing the need for transplantation in both acute and chronic liver failure, and potentially allowing more patients with primary or metastatic liver cancer to be treated successfully. Recent fate-mapping studies in mice have provided strong evidence that, in most murine models of liver injury and regeneration, restoration of liver mass occurs predominantly through self-duplication of hepatocytes.^1^, ^2^ Hence, identifying targets that promote proliferation and expansion of the preexistent hepatocyte population represents an attractive therapeutic approach to drive liver regeneration.

Transforming growth factor (TGF)-β has pleiotropic roles in liver disease. In addition to its role as a major proinflammatory cytokine,^3^ TGF-β is also a potent repressor of hepatocyte proliferation.^4^, ^5^, ^6^, ^7^ Therefore, in principle, TGF-β inhibition appears an attractive therapeutic strategy to promote hepatocyte proliferation and liver regeneration. An ideal therapy would target TGF-β with precision, allowing hepatocytes to escape the mito-inhibitory effects of TGF-β, while not abrogating the positive effects of TGF-β on extracellular matrix production and vascular remodeling during the regenerative process.^8^, ^9^ Furthermore, pan–TGF-β blockade may result in a number of unwanted, off-target effects, such as induction of autoimmunity and hepatocarcinogenesis.^10^, ^11^, ^12^ Therefore, a more nuanced, selective approach that targets the TGF-β pathway to promote liver regeneration is required.

TGF-β is predominantly stored within the extracellular matrix in a latent state, and much of the regulation of TGF-β function results from precise, temporally and spatially restricted, extracellular activation of this latent complex.^13^ The αv integrins, transmembrane heterodimeric proteins comprising an αv subunit and one of five β subunits, bind to an arginine-glycine-aspartate (RGD) motif present on the tip of an exposed loop within the latency-associated peptide that maintains TGF-β in an inactive state.^14^ All five αv integrins have been shown to interact with the RGD motif present in the latency-associated peptide.^15^, ^16^, ^17^, ^18^, ^19^ This integrin–RGD interaction, in the presence of mechanical force supplied by the integrin-expressing cell, enables the release of the active TGF-β homodimer.^20^

Inhibition of myofibroblast αv integrins in mice reduces fibrosis in multiple organs via a reduction in TGF-β activation.^21^ Furthermore, combined global knockout of integrins αvβ6 and αvβ8 phenocopies the developmental effects of loss of TGF-β–1 and –3.^22^ In the liver, expression of integrin αvβ6 appears restricted to activated cholangiocytes, transitional hepatocytes, and oval cells during biliary and portal fibrosis.^23^, ^24^ Conversely, αvβ8 expression by hepatic cell types has not been well characterized. Integrin αvβ8 has been shown to play an important role in TGF-β activation in other systems, including dendritic cells,^25^, ^26^, ^27^ regulatory T cells,^28^ neuroepithelium,^29^ and in fibroinflammatory airway disease.^30^ Further, integrin αvβ8 inhibits proliferation of lung epithelium via TGF-β activation.^31^ Therefore, given the important role of αvβ8 in mediating TGF-β activation in other organ systems and pathologic processes, we investigated the role of hepatocyte integrin αvβ8 in the context of liver regeneration. We hypothesized that depletion of integrin αvβ8 from hepatocytes would reduce local activation of TGF-β and would result in increased hepatocyte proliferation and accelerated liver regeneration after liver injury.

## Materials and Methods

### Mice

Albumin-Cre (*Alb-Cre*) mice^32^ were obtained from The Jackson Laboratory (Bar Harbor, ME), crossed with *Itgb8*^*flox/flox*^ mice^33^ obtained from Louis F. Reichardt (University of California, San Francisco, San Francisco, CA), and the resulting *Itgb8*^*flox/flox*^*;Alb-Cre* mice were maintained on a C57BL/6 background. *Pdgfrb-Cre* mice (also on a C57BL/6 background) were obtained from Ralf H. Adams (Max Planck Institute for Molecular Biomedicine and University of Münster, Münster, Germany).^34^ Mice used for all experiments were 8 to 16 weeks old and housed under specific pathogen–free conditions in the Animal Barrier Facility of the University of California, San Francisco, or the University of Edinburgh, UK. Genotyping of all mice was performed by PCR. Sample size was determined statistically before experimentation. Age- and sex-matched littermate controls were used for all experiments. Investigators (S.N.G., K.P.M., K.H., K.S., M.C.D., E.E.M.S., and N.C.H.) were blinded to mouse genotype, and experimental order was decided randomly. All experimental animal procedures were approved by the Institutional Animal Care and Use Committee of the University of California, San Francisco, or performed in accordance with the UK Home Office regulations.

### Two-Thirds Partial Hepatectomy

Two-thirds of the liver was surgically removed under isoflurane anesthesia as previously described.^35^ All surgeries were performed in the first half of the day. To label proliferating hepatocytes, 5-bromo-2′-deoxyuridine (BrdU; 10280879001; Sigma-Aldrich, Gillingham, UK) was injected 2 hours before liver harvest (100 mg/kg intraperitoneally). Mice and livers were weighed at harvest to calculate liver-to-body weight ratio.

### Hepatocarcinogenesis Model

Male mice were injected with diethylnitrosamine (25 mg/kg intraperitoneally; Sigma-Aldrich) at 12 to 14 days. Mice were sacrificed at 40 weeks, and macroscopic tumors were counted and measured.

### Liver Biochemistry

Whole blood was collected immediately after death and allowed to clot, and serum was obtained by centrifugation (9391 × *g* for 5 minutes twice). Samples were frozen at −20°C pending analysis. Serum albumin, total bilirubin, alanine transaminase, and alkaline phosphatase measurements were determined by using commercial kits (Alpha Laboratories, Eastleigh, UK, for albumin, bilirubin, and alanine transaminase; Randox Laboratories, Crumlin, County Antrim, UK, for alkaline phosphatase) adapted for use on a Cobas Fara centrifugal analyzer (Roche Diagnostics, Welwyn Garden City, UK).

### Immunohistochemistry

Liver samples were fixed overnight at room temperature in either methacarn (60% methanol, 30% chloroform, 10% acetic acid) for BrdU immunohistochemistry (IHC) or 10% neutral-buffered formalin. Samples were then paraffin-embedded before sectioning.

No image processing was performed before quantitative analysis. Images presented in figures were contrast-enhanced by adjusting intensity minima and maxima. Images to be compared were processed identically and in a manner that preserved the visibility of dim and bright structures in the original image.

### BrdU IHC

Endogenous peroxidases were quenched with 0.3% H_2_O_2_ in methanol, followed by consecutive 10-minute incubation steps with 0.1% trypsin (T7168; Sigma-Aldrich), warm 1.8 mol/L HCl, and 0.1 mol/L sodium tetraborate decahydrate (S9640; Sigma-Aldrich). Blocking and subsequent incubation steps used the Mouse on Mouse Elite Peroxidase Kit (PK2200; Vector Laboratories, Peterborough, UK). Primary antibody was mouse anti-BrdU (dilution 1:40; M0744; Dako, Agilent Technologies, Cheadle, UK). Detection was performed by using the Elite Vectastain ABC kit (PK7100; Vector Laboratories) and 3,3′-diaminobenzidine (K3468; Dako) before counterstaining, dehydration, and mounting. For each liver sample, approximately 3000 hepatocytes were counted to calculate the percentage of proliferating hepatocytes.

### GR1 (neutrophil)/F4/80 (Kupffer cell)/PDGFRβ (hepatic stellate cell) IHC

Antigen retrieval was performed with Tris-EDTA [platelet-derived growth factor receptor (PDGFR)β only], endogenous peroxidases were quenched with 3% H_2_O_2_, Avidin/Biotin block was applied (SP-2001; Vector Laboratories) before blocking with 20% goat serum (GR1/PDGFRβ) or rabbit serum (F4/80). Primary antibodies were applied for 2 hours at room temperature (PDGFRβ, dilution 1:500; ab32570; Abcam, Cambridge, UK) or overnight at 4°C (GR1, dilution 1:750; MAB1037; R&D, Abingdon, UK; F4/80, dilution 1:200; ab6640; Abcam). Secondary antibody (PDGFRβ–biotinylated goat anti-rabbit, dilution 1:1000; BA-1000; Vector Laboratories; GR1–biotinylated goat anti-rat, dilution 1:1000; BA-9401; F4/80–biotinylated rabbit anti-rat, dilution 1:200; BA-4001) was applied for 30 minutes at room temperature. Detection was performed by using the Elite Vectastain ABC kit and 3,3′-diaminobenzidine, before counterstaining, dehydration, and mounting. For each sample, 10 sequential fields were acquired at ×20 magnification, and the percentage of positive staining was calculated by using FIJI.^36^

### β8 Integrin Subunit/Cleaved Caspase-3 IHC

Antigen retrieval was performed with Tris-EDTA (β8 integrin subunit) or sodium citrate (cleaved caspase-3), endogenous peroxidases were quenched with 3% H_2_O_2_ before blocking with 20% horse serum. Primary antibody (β8 integrin subunit, dilution 1:500; ab80673; Abcam; Cleaved Caspase-3, dilution 1:1000; 9664; Cell Signaling Technology, Leiden, The Netherlands) was applied overnight at 4°C. Detection was performed by using the ImmPRESS Polymerized Reporter Enzyme Staining System (MP7401; Vector Laboratories) and 3,3′-diaminobenzidine before counterstaining, dehydration, and mounting.

### Hematoxylin and Eosin Staining

Sections were baked at 55°C overnight before de-waxing and rehydration. Slides were then placed in Harris Hematoxylin (Thermo Fisher Scientific, Paisley, UK) for 5 minutes. After washing, slides were placed in 1% acid alcohol for 5 seconds, followed by Scott's tap water for 2 minutes. Slides were then transferred to Eosin Y solution (Thermo Fisher Scientific) for 2 minutes, followed by washing, dehydration, and mounting. For quantification of mitotic figures, a minimum of 1000 hepatocytes were counted per sample.

### Primary Mouse Hepatocyte Isolation

Primary mouse hepatocytes were isolated by retrograde perfusion of the liver with Liver Perfusion Medium (Thermo Fisher Scientific), followed by Liver Digest Medium (Thermo Fisher Scientific) at 37°C. When hepatocytes were visually dispersed within the liver capsule, the liver was removed to a sterile dish and minced with scissors to release the crude cell isolate. The cells were then suspended in Dulbecco’s modified Eagle’s medium/F-12 (Thermo Fisher Scientific) and pelleted twice. Hepatocytes were purified from the washed pellets by resuspension in culture medium and centrifugation through 50% equilibrated Percoll (GE Healthcare Life Sciences, Little Chalfont, UK).

### Standard Primary Hepatocyte Culture

Primary hepatocytes were isolated as described in the section above, resuspended in low-serum medium [Dulbecco’s modified Eagle’s medium (Thermo Fisher Scientific), 2.5% fetal bovine serum (Thermo Fisher Scientific), 2% l-glutamine (Thermo Fisher Scientific), 1% penicillin streptomycin (Thermo Fisher Scientific)], and plated onto collagen-coated wells (Collagen Type I; Millipore, Watford, UK) in a 6-well plate at a density of 500,000 cells per well. Either β8 integrin subunit blocking antibody^26^ or nonbinding control antibody were added at 20 μg/mL, and samples were incubated for 24 hours at 37°C in 5% CO_2_. Wells were then washed with phosphate-buffered saline (PBS), and cells were lysed as described in the following section.

### RT-qPCR

RNA was isolated from whole mouse liver, primary hepatocytes, or liver sinusoidal endothelial cells by using a RNeasy Mini Kit (whole liver, hepatocytes) or Rneasy Plus Micro Kit (liver sinusoidal endothelial cells; Qiagen, Manchester, UK). cDNA transcription and real-time quantitative PCR (qPCR) were performed by using a SYBR-GreenER Two-Step qRT-PCR kit (Invitrogen, Thermo Fisher Scientific) or QuantiTect Reverse Transcription and SYBR Green PCR Kits (Qiagen). Samples were amplified on an ABI 7900HT thermocycler (Applied Biosystems, Thermo Fisher Scientific) and normalized to *Actb* and/or *Gapdh* expression. Primers used were as follows: *Actb*, 5′TGTTACCAACTGGGACGACA-3′ (forward) and 5′-GGGGTGTTGAAGGTCTCAAA-3′ (reverse); *Itgb8*, 5′-CTGAAGAAATACCCCGTGGA-3′ (forward) and 5′-ATGGGGAGGCATACAGTCT-3′ (reverse). Quantitect Primer Assays (249990; Qiagen) were used for the following genes: *Ccna2* (QT00102151), *Ccnb1* (QT00152040), *Ccnd1* (QT00154595), *Ccne1* (QT00103495), *Cdkn1a* (QT00137053), *Cdkn1b* (QT01058708), *Gapdh* (QT01658692), and *Plat* (QT00133630).

To assess TGF-β signaling, a custom RT^2^ Profiler PCR array (330171; Qiagen) was designed which contained primer sequences for the genes shown in Supplemental Table S1. RNA was isolated after primary hepatocyte culture as described in the section above and reversed transcribed by using the RT^2^ First Strand Kit (330401; Qiagen). qPCR was performed by using RT^2^ SYBR Green ROX qPCR Mastermix (330522; Qiagen) on an ABI 7900HT thermocycler, normalized to *Actb* and *Gapdh* expression.

### Hepatocyte Adhesion Assay

Adhesion was assessed by using a colorimetric ECM Cell Adhesion Array Kit (ECM540; Millipore) according to the manufacturer’s instructions. Primary mouse hepatocytes were isolated as described above, plated in triplicate at 50,000 cells per well, and incubated for 2 hours at 37°C in 5% CO_2_. Absorbance was measured at 570 nm by using a Synergy HT microplate reader (BioTek, Swindon, UK). Relative absorbance was calculated by standardizing to absorbance in the Collagen I well, before calculation of mean relative absorbance for each extracellular matrix protein for each sample.

### Hepatocyte Proliferation Assay

Primary mouse hepatocytes were isolated as above and plated at 10,000 cells per well in 24-well plates (353847; Primaria; Corning, St David’s Park, UK) in Dulbecco’s modified Eagle’s medium/F-12 supplemented with 15 mmol/L HEPES (H3375; Sigma-Aldrich), 10% fetal bovine serum, 1% insulin-transferrin-selenium (41400-045; Thermo Fisher Scientific), and 1% penicillin streptomycin. Cells were allowed to adhere for 4 hours before washing with PBS. Cells were then cultured for 48 hours in a low-serum version of the plating medium, containing only 0.5% fetal bovine serum. The β8 integrin subunit blocking antibody or nonbinding control antibody was added at 20 μg/mL. Growth factors [hepatocyte growth factor (HGF), PHG0254, and epidermal growth factor (EGF), PMG8044; Thermo Fisher Scientific] were added at 40 ng/mL. Culture medium, antibodies, and growth factors were refreshed at 24 hours, at which time 10 μmol/L EdU (5-ethynyl-2′-deoxyuridine; C10640; Thermo Fisher Scientific) was added. After the 48-hour culture period, cells were washed with PBS-BSA [PBS supplemented with 1% bovine serum albumin (BSA); A8806; Sigma-Aldrich] and then fixed by using 4% paraformaldehyde in PBS for 15 minutes at room temperature.

Proliferating hepatocytes were detected by using the Click-iT Plus EdU Alexa Fluor 647 Imaging Kit (C10640; Thermo Fisher Scientific). Briefly, fixed cells were washed with PBS-BSA and incubated in 0.5% Triton X-100 (T8787; Sigma-Aldrich) in PBS for 20 minutes at room temperature. After washing, the Click-iT Plus reaction cocktail was added, and cells were incubated for 30 minutes at room temperature and protected from light. The cells were washed again and then incubated in 5 μg/mL Hoechst 33342 (Thermo Fisher Scientific) for 30 minutes at room temperature and protected from light. Finally, cells were washed with PBS and imaged. Imaging was performed by using an LSM780 confocal microscope system (Carl Zeiss Ltd, Cambridge, UK). Tiled images were acquired, with three non-overlapping areas of 18 μm^2^ imaged per well. Imaris version 8.4.1 (Bitplane AG, Zurich, Switzerland) was used to identify the total (Hoechst-positive) nuclei number and the number of EdU-positive nuclei, and the percentage of proliferating nuclei was calculated.

### Whole Liver Microarray

Sample preparation, labeling, and array hybridizations were performed by using the Agilent GE 4 × 44 Mouse microarray platform (Agilent Technologies, Palo Alto, CA). Total RNA quality was assessed by using a Pico Chip on an Agilent 2100 Bioanalyzer, and RNA was amplified and labeled with cyanine 3–cytidine-5′-triphosphate by using the Agilent Technologies low RNA input fluorescent linear amplification kits according to the manufacturer’s protocol. Labeled cRNA was assessed by using the Nanodrop ND-100 (Nanodrop Technologies, Inc., Wilmington DE), and equal amounts of cyanine 3-labeled target were hybridized to Agilent whole mouse genome 4 × 44K Ink-jet arrays (G4122F; Agilent Technologies). Hybridizations were performed for 14 hours, according to the manufacturer’s protocol. Arrays were scanned by using the Agilent Technologies microarray scanner and raw signal intensities were extracted with Agilent Feature Extraction version 10.5 software. Raw data are accessible at the Gene Expression Omnibus repository (*http://www.ncbi.nlm.nih.gov/geo*; accession number GSE111591).

### Human Liver Tissue

De-identified sections of uninjured and fibrotic human liver tissue were provided by the Lothian NRS Bioresource with approval from Tissue Governance. Samples of acetaminophen-injured human liver tissue were obtained as part of the Pathophysiology of Acute Liver Injury study. This study was approved by the Scotland A Research Ethics Committee and National Health Service Lothian Research and Development.

### Statistical Analysis

The statistical significance of differences between groups was calculated with a two-tailed *t*-test or *U*-test as appropriate. Differences with a *P* value < 0.05 were considered statistically significant. PCR data obtained for individual genes were log-transformed before analysis, and a Bonferroni correction was applied to account for multiple testing. PCR array data were standardized as previously reported,^37^ to identify genes in test samples with a 95% CI for standardized relative fold change that did not overlap 1 (the value assigned to the fold change for the same gene in control samples).

For microarray analysis, differential gene expression was examined with the R package limma version 3.32.7.^38^ Quality control was performed by identifying outliers in the log2 intensity between arrays and comparison of multidimensional scaling of distances between microarray expression profiles. Background correction was conducted according to the *normexp* method, and the data were normalized by using the *quantile* normalization method.^39^, ^40^ A two-way analysis of variance linear model was fitted to the comparison to estimate the mean M values and to calculate moderated *t*-statistic, B statistic, false discovery rate, and *P* value for each gene for the comparison of interest. Adjusted *P* values were produced by the method proposed by Holm.^41^ Gene Ontology (GO) analysis was performed with the R package topGO version 2.28.0,^42^ and the *elim* algorithm, combined with the Fisher exact test, was used to calculate the enrichment scores for each of the GO terms.

## Results

### Depletion of Hepatocyte Integrin αvβ8 Leads to Increased Hepatocyte Proliferation and Accelerated Liver Regeneration

**Figure 1:**
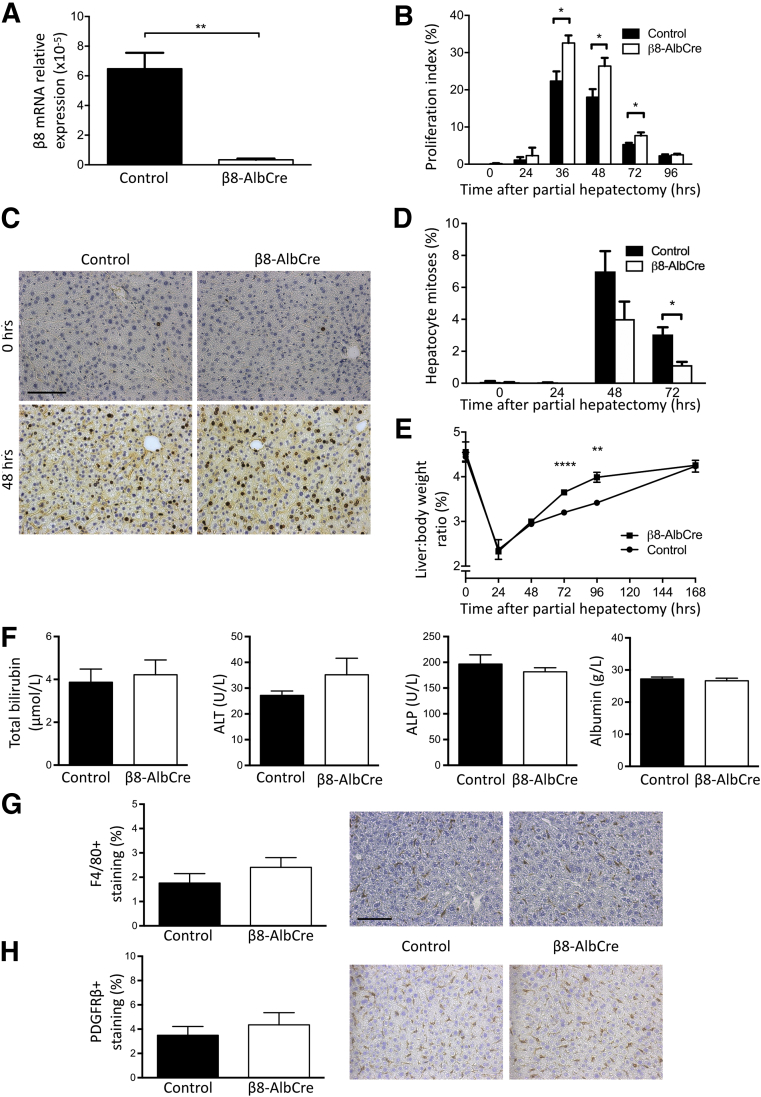
Genetic depletion of hepatocyte integrin αvβ8 accelerated liver regeneration. **A:** Real-time quantitative PCR of *Itgb8* expression in hepatocytes isolated from control and *Itgb8*^*flox/flox*^*;Alb-Cre* (β8-AlbCre) mice. **B:** Quantitation of 5-bromo-2′-deoxyuridine (BrdU)^+^ hepatocyte nuclei in control and β8-AlbCre mice after partial hepatectomy. **C:** Representative images from BrdU immunostaining of liver sections from control and β8-AlbCre mice at 0 and 48 hours after partial hepatectomy. **D** and **E:** Quantitation of hepatocyte mitoses (**D**) and liver-to-body weight ratio (**E**) in control and β8-AlbCre mice after partial hepatectomy. **F:** Serum biochemistry [total bilirubin, alanine transaminase (ALT), alkaline phosphatase (ALP), albumin] from uninjured control and β8-AlbCre mice. **G** and **H:** Quantification and representative images from F4/80 (Kupffer cell; **G**) and platelet-derived growth factor receptor (PDGFR)β (hepatic stellate cell; **H**) immunostaining of liver tissue from uninjured control and β8-AlbCre mice. Data are expressed as means ± SEM. *n* = 3 control and β8-AlbCre mice (**A**); *n* = 3 to 6 control and β8-AlbCre mice per time point (**B**–**E**); *n* = 6 control and β8-AlbCre mice (**F**–**H**). ^∗^*P* < 0.05, ^∗∗^*P* < 0.01, and ^∗∗∗∗^*P* < 0.0001. Scale bars: 100 μm (**C** and **G**).

**Supplemental Figure S1:**
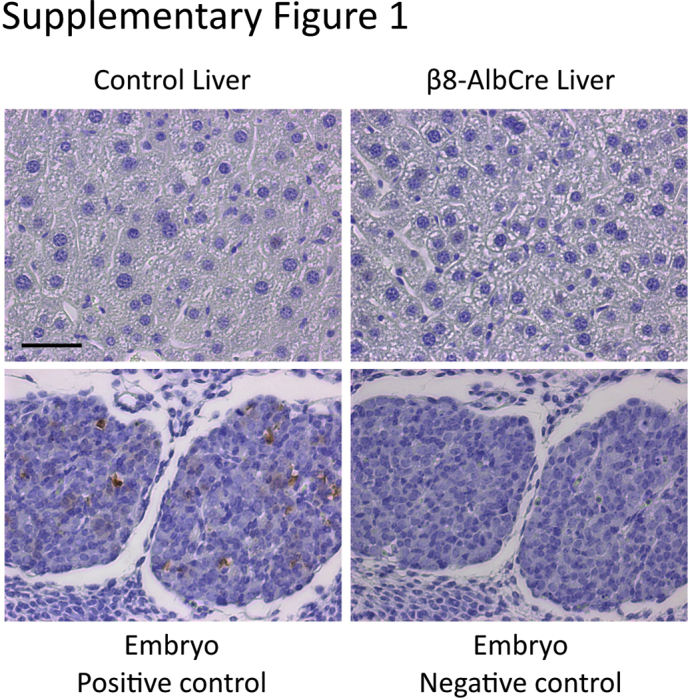
Hepatocyte integrin αvβ8 depletion did not increase apoptosis in the liver after partial hepatectomy. Representative images of cleaved caspase-3 immunostaining of liver tissue from control and *Itgb8*^*flox/flox*^*;Alb-Cre* (β8-AlbCre) mice at 5 days after partial hepatectomy (upper). Positive and negative staining controls of mouse embryo tissue are also shown (lower). *n* = 5 control and β8-AlbCre mice. Scale bar = 50 μm.

To deplete integrin αvβ8 in hepatocytes, *Itgb8*^*flox/flox*^*;Alb-Cre* mice were generated.^32^, ^33^ Primary hepatocytes were isolated from *Itgb8*^*flox/flox*^*;Alb-Cre* mice and Cre-negative littermate controls. qPCR for *Itgb8* confirmed expression in control hepatocytes and successful depletion in hepatocytes isolated from *Itgb8*^*flox/flox*^*;Alb-Cre* mice (Figure 1A). Assessment of hepatocyte proliferation after two-thirds partial hepatectomy showed significantly increased proliferation in *Itgb8*^*flox/flox*^*;Alb-Cre* mice at 36, 48, and 72 hours after liver injury compared with control mice (Figure 1, B and C). This increased hepatocyte proliferation was not followed by an increase in hepatocyte apoptosis at day 5 after partial hepatectomy, when liver regeneration was nearing completion in the *Itgb8*^*flox/flox*^*;Alb-Cre* mouse (Supplemental Figure S1). Interestingly, the proportion of hepatocyte mitoses (identified morphologically) was decreased in *Itgb8*^*flox/flox*^*;Alb-Cre* mice at 72 hours after liver injury compared with control mice (Figure 1D). However, liver-to-body weight ratio was significantly increased in *Itgb8*^*flox/flox*^*;Alb-Cre* mice at 72 and 96 hours after partial hepatectomy, demonstrating that the increase in hepatocyte proliferation in *Itgb8*^*flox/flox*^*;Alb-Cre* mice detected by BrdU IHC resulted in accelerated restoration of liver mass compared with control mice (Figure 1E).

### Depletion of Hepatocyte Integrin αvβ8 Does Not Alter Baseline Hepatocyte Proliferation or Subsequent Inflammatory Phenotype

**Figure 2:**
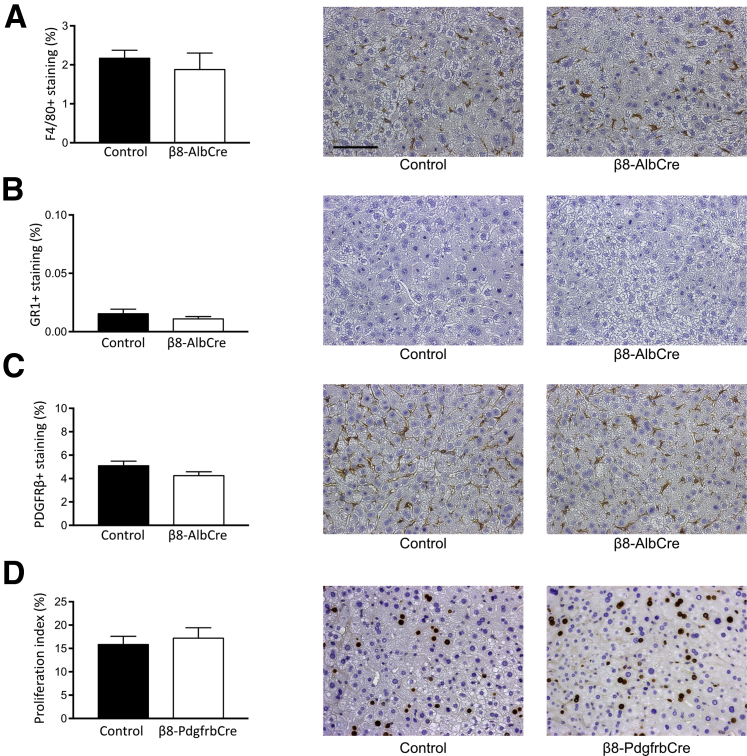
Depletion of hepatocyte integrin αvβ8 did not alter inflammatory phenotype after partial hepatectomy. **A**–**C:** Quantification and representative images from F4/80 (Kupffer cell; **A**), GR1 (neutrophil; **B**), and platelet-derived growth factor receptor (PDGFR)β (hepatic stellate cell; **C**) immunostaining of liver tissue from control and *Itgb8*^*flox/flox*^*;Alb-Cre* (β8-AlbCre) mice at 48 hours after partial hepatectomy. **D:** Quantitation of 5-bromo-2′-deoxyuridine (BrdU)^+^ hepatocyte nuclei and representative images of BrdU immunostaining of liver tissue from control and *Itgb8*^*flox/flox*^*;Pdgfrb-Cre* (β8-PdgfrbCre) mice at 48 hours after partial hepatectomy. Data are expressed as means ± SEM. *n* = 5 control and β8-AlbCre mice (**A**–**C**); *n* = 4 and 8 control and β8-PdgfrbCre mice (**D**). Scale bar = 100 μm.

**Supplemental Figure S2:**
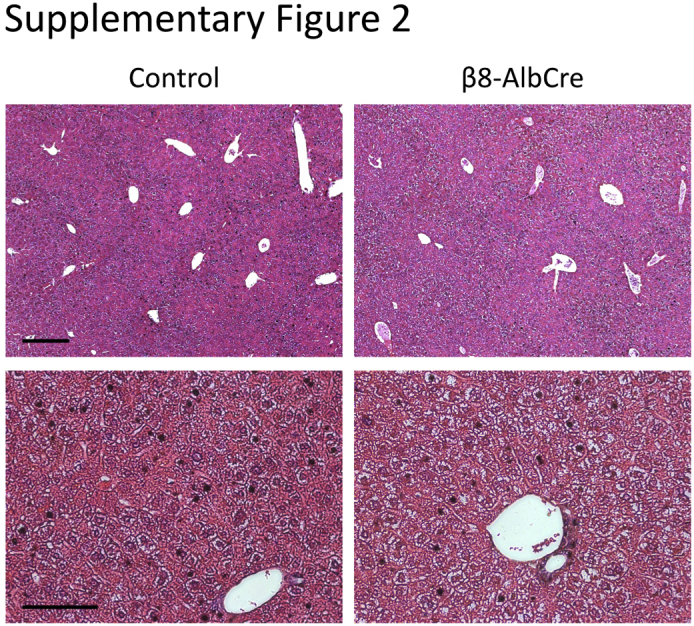
Hepatocyte integrin αvβ8 depletion did not alter liver architecture. Representative images of hematoxylin and eosin staining of liver tissue from uninjured control and *Itgb8*^*flox/flox*^*;Alb-Cre* (β8-AlbCre) mice. Scale bars: 250 μm (**top row**); 100 μm **(bottom row**).

Because integrin αvβ8 is able to activate TGF-β, a well-characterized suppressor of epithelial proliferation, the effect of genetic depletion of hepatocyte αvβ8 on baseline hepatocyte proliferation or liver-to-body weight ratio was assessed. Hepatocyte BrdU incorporation and mitoses and liver-to-body weight ratio were measured in uninjured *Itgb8*^*flox/flox*^*;Alb-Cre* mice and control mice (Figure 1, B, D, and E), and no difference was found in any of these variables between groups. Furthermore, no difference was found in baseline liver biochemistry, hepatic morphology, or resident nonparenchymal cell populations [Kupffer cells and hepatic stellate cells (HSCs)] between uninjured *Itgb8*^*flox/flox*^*;Alb-Cre* mice and control mice (Figure 1, F–H, Supplemental Figure S2). After partial hepatectomy, no difference was found in hepatic inflammation (Kupffer cells or neutrophils) or HSC immunostaining (Figure 2, A–C). This suggested that the increased liver regeneration observed after partial hepatectomy in *Itgb8*^*flox/flox*^*;Alb-Cre* mice was not due to differences in degree of initial injury or the subsequent inflammatory response.

### Depletion of HSC Integrin αvβ8 Does Not Lead to Increased Hepatocyte Proliferation

Integrin αvβ8 is also expressed on HSCs.^21^ Because HSCs have been shown to play an important regulatory role in liver regeneration,^43^, ^44^ mice in which integrin αvβ8 had been depleted from HSCs (*Itgb8*^*flox/flox*^*;Pdgfrb-Cre*) were used to examine the role of HSC integrin αvβ8 during liver regeneration. After two-thirds partial hepatectomy, no significant difference was found in hepatocyte proliferation between *Itgb8*^*flox/flox*^*;Pdgfrb-Cre* mice and control mice (Figure 2D). Liver sinusoidal endothelial cells have also been shown to play a key role in liver regeneration.^45^, ^46^, ^47^ However, integrin αvβ8 expression was not observed in liver sinusoidal endothelial cells by qPCR.

### Assessment of Hepatic Cell Cycle Genes after Depletion of Hepatocyte Integrin αvβ8 and Partial Hepatectomy

**Figure 3:**
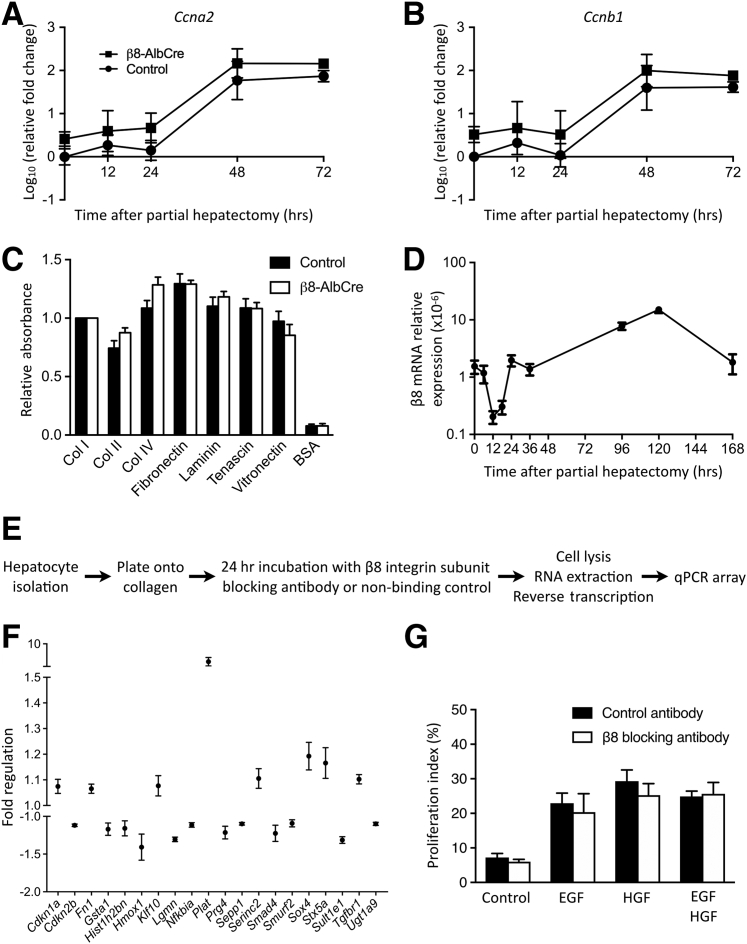
Investigation of the mechanisms mediating the pro-regenerative effect of hepatocyte integrin αvβ8 depletion. **A** and **B:** Whole liver expression of cell cycle genes *Ccna2* (**A**) and *Ccnb1* (**B**) from control and *Itgb8*^*flox/flox*^*;Alb-Cre* (β8-AlbCre) mice after partial hepatectomy. **C:** Isolated hepatocytes from control and β8-AlbCre mice were tested in a colorimetric extracellular matrix adhesion assay. **D:** Whole liver expression of *Itgb8* after partial hepatectomy. **E:** Schematic of experimental design to test the effect of a β8 integrin subunit blocking antibody on hepatocyte expression of transforming growth factor-β–responsive genes. **F:** Fold regulation of genes from the real-time quantitative PCR (qPCR) array with a detectable change in hepatocyte expression after culture with β8 integrin subunit blocking antibody. **G:** Proliferation of primary hepatocytes cultured for 48 hours with β8 integrin subunit blocking antibody or control antibody in culture medium (Control) and with addition of either epidermal growth factor (EGF) or hepatocyte growth factor (HGF) or with both. Data are expressed as means ± SEM. *n* = 4 to 6 control and β8-AlbCre mice per time point (**A** and **B**); *n* = 4 control and β8-AlbCre mice (**C**); *n* = 3 to 6 mice per time point (**D**); *n* = 3 mice (**F** and **G**). BSA, bovine serum albumin; Col, collagen.

**Supplemental Figure S3:**
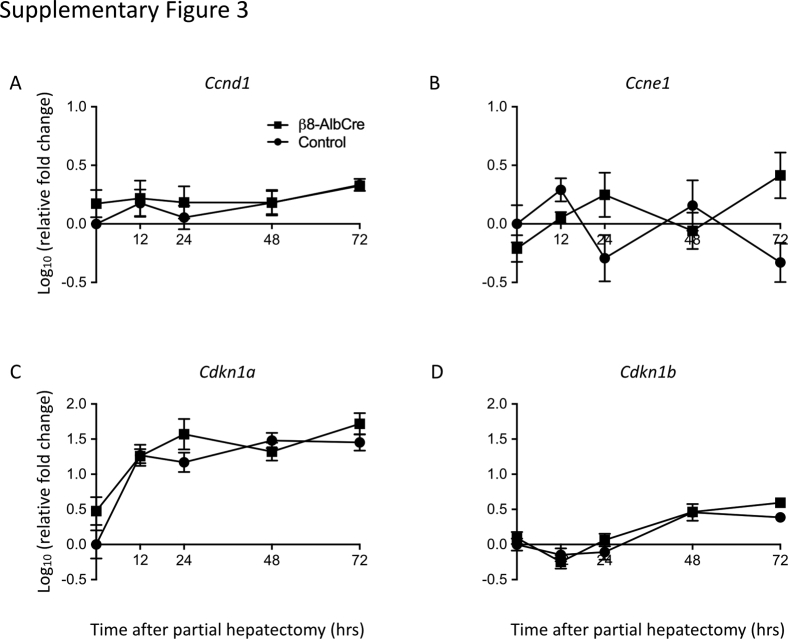
Assessment of hepatic cell cycle genes after depletion of hepatocyte integrin αvβ8 and partial hepatectomy. **A**–**D:** Whole liver expression of *Ccnd1* (**A**), *Ccne1* (**B**), *Cdkn1a* (**C**), and *Cdkn1b* (**D**) from control and *Itgb8*^*flox/flox*^*;Alb-Cre* (β8-AlbCre) mice after partial hepatectomy. Data are expressed as means ± SEM. *n* = 4 to 6 control and β8-AlbCre mice per time point.

To examine whether depletion of hepatocyte integrin αvβ8 might have a direct effect on the cell cycle, the expression of genes with key roles in cell cycle regulation was measured at multiple time points after partial hepatectomy. Overall, partial hepatectomy resulted in expected changes in gene expression in whole liver from both *Itgb8*^*flox/flox*^*;Alb-Cre* mice and control mice (Figure 3, A and B, Supplemental Figure S3). A trend toward increased expression of *Ccna2* and *Ccnb1* was found in *Itgb8*^*flox/flox*^*;Alb-Cre* mice compared with control mice; however, this trend did not reach statistical significance at any time point (Figure 3, A and B). Analysis of other cell cycle–related genes (*Ccnd1*, *Ccne1*, *Cdkn1a*, *Cdkn1b*) showed no difference between *Itgb8*^*flox/flox*^*;Alb-Cre* mice and control mice (Supplemental Figure S3).

### Depletion of Integrin αvβ8 on Hepatocytes Does Not Alter Adhesion to Multiple Matrix Proteins Present in Normal and Regenerating Liver

Integrin αvβ8 binds extracellular matrix ligands such as vitronectin, fibronectin, collagen IV, and fibronectin.^48^, ^49^ To test whether depletion of integrin αvβ8 on hepatocytes altered adhesion to cell matrix proteins present in normal and regenerating liver, an *in vitro* cell adhesion assay with multiple different matrix substrates was used. No difference was found in adhesion between *Itgb8*^*flox/flox*^*;Alb-Cre* and control hepatocytes across all seven matrix proteins tested (Figure 3C), suggesting that altered hepatocyte adhesion was not responsible for the pro-regenerative phenotype observed in *Itgb8*^*flox/flox*^*;Alb-Cre* mice.

### Inhibition of Integrin αvβ8 Modulates TGF-β-Responsive Genes in Hepatocytes

Integrin αvβ8 has previously been shown to play a key role in the activation of latent TGF-β,^25^, ^26^, ^27^, ^28^, ^29^, ^30^, ^31^ a potent inhibitor of hepatocyte proliferation.^5^, ^6^, ^7^ Therefore, we hypothesized that depletion of hepatocyte integrin αvβ8 might promote hepatocyte proliferation through modulation of TGF-β signaling pathways. The time course of hepatic *Itgb8* expression after partial hepatectomy supported a role for integrin αvβ8 as a suppressor of hepatocyte proliferation during liver regeneration (Figure 3D). Hepatic *Itgb8* expression fell markedly in the 24 hours immediately after partial hepatectomy, and this down-regulation appeared to be permissive for hepatocyte proliferation. At the same time as the liver approached full restoration of its functional mass at 5 days after partial hepatectomy, hepatocyte *Itgb8* expression peaked at 10 times baseline expression, consistent with a role for integrin αvβ8 as a brake on hepatocyte proliferation (Figure 3D).

**Supplemental Figure S4:**
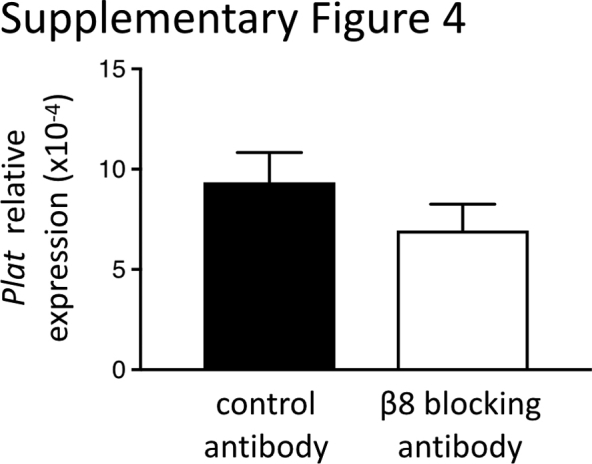
Treatment of hepatocytes isolated from *Itgb8*^*flox/flox*^*;Alb-Cre* mice with β8 integrin subunit blocking antibody did not alter *Plat* expression. Data are expressed as means ± SEM. *n* = 3 β8-AlbCre mice.

Detecting the modulation of TGF-β activation in the hepatocyte regenerative niche is challenging, because it is not possible to measure the levels of active TGF-β in tissue directly. Therefore, an experiment was designed to examine how inhibition of integrin αvβ8 might modulate TGF-β–responsive genes in primary mouse hepatocytes (Figure 3E). First, a custom qPCR array was designed, containing 87 genes either shown to be responsive to TGF-β signaling in hepatocytes^50^ or comprising components of the TGF-β pathway (Supplemental Table S1). Primary murine hepatocytes were isolated from wild-type mice and plated onto collagen in the presence of either a β8 integrin subunit blocking antibody or a nonbinding control antibody.^26^ After incubation for 24 hours, hepatocytes were lysed, RNA was extracted, and gene expression was quantified by using the custom qPCR array. Data were then standardized as previously described by Willems et al.^37^ After culture with β8 integrin subunit blocking antibody, 20 genes in the qPCR array were found to have a detectable change in expression with a 95% CI, which did not overlap control values (Figure 3F). Of these, 12 genes showed >10% up- or down-regulation compared with controls, with 10 of 12 responding as predicted. *Plat*, encoding tissue plasminogen activator (tPA), showed the greatest up-regulation. Hepatocyte expression of *Plat* has been shown to decrease in the presence of TGF-β,^50^ whereas expression increased threefold in wild-type hepatocytes treated with β8 integrin subunit blocking antibody. Increased expression of *Plat* was not observed when hepatocytes from *Itgb8*^*flox/flox*^*;Alb-Cre* mice were treated with β8 integrin subunit blocking antibody, suggesting the observed response was specific to β8 integrin subunit inhibition (Supplemental Figure S4). Conversely, hepatocyte expression of the TGF-β–responsive gene *Hmox1* (heme oxygenase 1) was down-regulated in the presence of β8 integrin subunit blocking antibody. These data indicated that inhibition of integrin αvβ8 modulated TGF-β–responsive genes in hepatocytes, suggesting a possible mechanism through which integrin αvβ8 depletion promoted hepatocyte proliferation.

### Inhibition of Hepatocyte Integrin αvβ8 Does Not Alter the Proliferative Response to Mitogenic Growth Factors

HGF and EGF are key drivers of liver regeneration.^8^ To investigate whether integrin αvβ8 might have a role in regulating the hepatocyte response to these direct mitogens, the effect of β8 integrin subunit inhibition during *in vitro* proliferation of primary hepatocytes in response to EGF and HGF was examined. A robust increase in hepatocyte proliferation was achieved with addition of either EGF or HGF, or with both, compared with standard culture medium (Figure 3G). However, inhibition of integrin αvβ8 had no effect on the degree of *in vitro* hepatocyte proliferation. This suggested that the accelerated liver regeneration observed after hepatocyte integrin αvβ8 depletion did not occur via modulation of HGF or EGF downstream signaling pathways.

### Microarray Analysis of Whole Liver from Control and *Itgb8*^*flox/flox*^*;Alb-Cre* Mice

**Figure 4:**
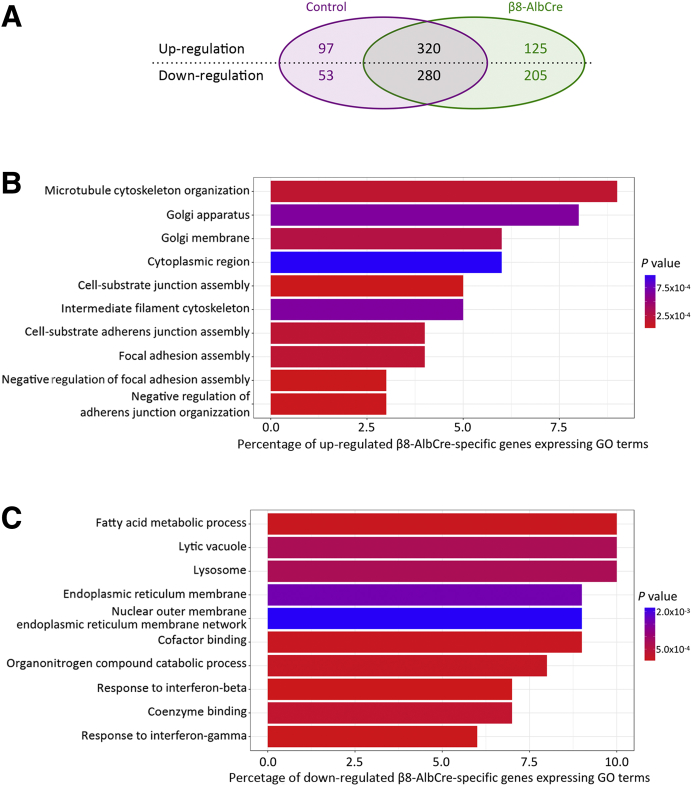
Microarray analysis of whole liver (uninjured and 24 hours after partial hepatectomy) from control and *Itgb8*^*flox/flox*^*;Alb-Cre* (β8-AlbCre) mice. **A:** Summary of the number of transcripts with significant changes in expression after partial hepatectomy in β8-AlbCre and control mice. **B** and **C:** The top 10 Gene Ontology (GO) terms enriched in those genes either up-regulated (**B**) or down-regulated (**C**) exclusively in β8-AlbCre mice after partial hepatectomy. *n* = 4 control and β8-AlbCre mice per group per time point.

To explore further the potential mechanisms by which inhibition of integrin αvβ8 increased hepatocyte proliferation and accelerated liver regeneration, global gene expression changes were examined by microarray analysis of whole liver. Samples were obtained from control and *Itgb8*^*flox/flox*^*;Alb-Cre* mice before and at 24 hours after partial hepatectomy. From 26,136 transcripts, 1080 showed statistically significant differential expression after partial hepatectomy. Of these, 330 occurred only in the *Itgb8*^*flox/flox*^*;Alb-Cre* mouse (Figure 4A). GO analysis was performed on the differentially expressed genes. The dominant GO terms enriched in genes up-regulated exclusively in *Itgb8*^*flox/flox*^*;Alb-Cre* mice after partial hepatectomy are shown in Figure 4B. Most terms related to cytoskeletal organization and cellular adhesion. Similarly, the dominant GO terms enriched in genes down-regulated exclusively in *Itgb8*^*flox/flox*^*;Alb-Cre* mice after partial hepatectomy are shown in Figure 4C. These terms were relatively nonspecific, relating to a range of intracellular metabolic processes.

Given the increased hepatocyte proliferation observed after partial hepatectomy in *Itgb8*^*flox/flox*^*;Alb-Cre* mice, changes in expression of genes known to regulate the cell cycle were specifically studied. However, when comparing samples after partial hepatectomy from *Itgb8*^*flox/flox*^*;Alb-Cre* mice and control mice, no significant difference in the expression of cell cycle genes was detected by microarray. This mirrored the findings of both our examination of cell cycle gene expression in whole liver before and after partial hepatectomy (Figure 3, A and B and Supplemental Figure S3) and the qPCR array in cultured hepatocytes treated with β8 integrin subunit blocking antibody, in which no consistent changes in gene expression were noted in the 10 cell cycle and proliferation genes included in the array (Supplemental Table S1).

### Depletion of Hepatocyte Integrin αvβ8 Does Not Increase Tumor Formation in a Mouse Model of HCC

**Figure 5:**
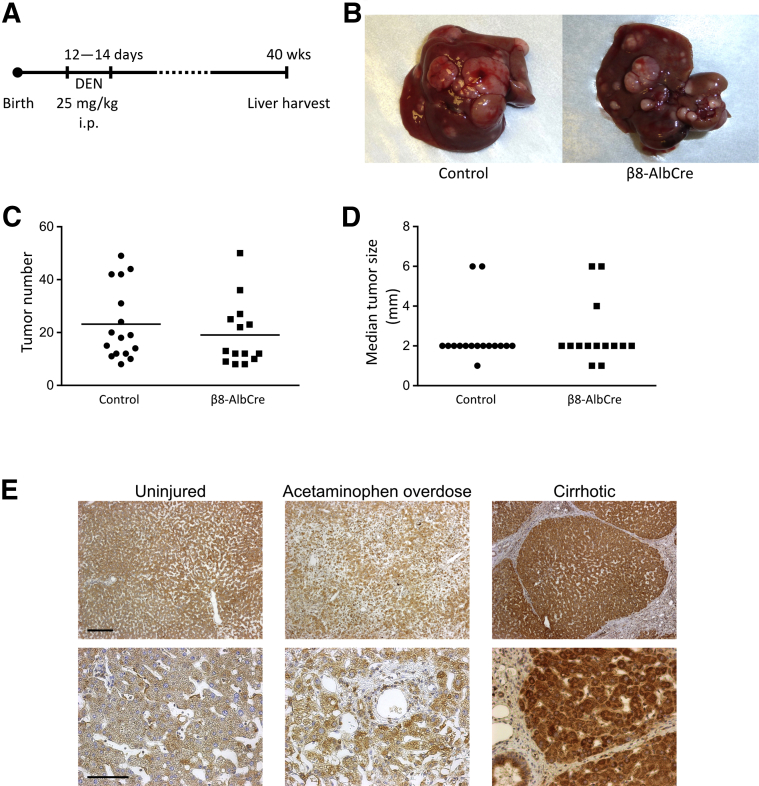
Depletion of hepatocyte integrin αvβ8 did not increase hepatocellular carcinoma (HCC) in mice, whereas human hepatocytes expressed integrin αvβ8 in acute and chronic liver disease. **A:** Schematic of mouse model of HCC. **B:** Representative images of livers from control and *Itgb8*^*flox/flox*^*;Alb-Cre* (β8-AlbCre) mice at harvest. **C** and **D:** Quantification of tumor number (**C**) and median tumor size (**D**) in control and β8-AlbCre mice at 40 weeks (**horizontal bar** indicates mean). **E:** Representative low- and high-power images of β8 integrin subunit immunostaining in uninjured human liver tissue, after acetaminophen overdose, or in cirrhosis. *n* = 16 and 14 control and β8-AlbCre mice (**C** and **D**); *n* = 5 uninjured human liver tissue samples (**E**); *n* = 5 acetaminophen overdose samples (**E**); *n* = 6 cirrhosis samples (**E**). Scale bars: 250 μm (**top row**; **E**); 100 μm (**bottom row**; **E**). DEN = diethylnitrosamine.

In addition to promoting hepatocyte proliferation, disruption of TGF-β signaling can accelerate the development of hepatocellular carcinoma (HCC) in mice after diethylnitrosamine administration.^12^ Because depletion of integrin αvβ8 on hepatocytes increased hepatocyte proliferation and accelerated liver regeneration after injury, and blockade of hepatocyte integrin αvβ8 *in vitro* modulated TGF-β-responsive genes, the possibility that this pro-proliferative phenotype might increase the risk of HCC development was also assessed. *Itgb8*^*flox/flox*^*;Alb-Cre* and control mice were injected with diethylnitrosamine at 12 to 14 days of age to induce HCC (Figure 5A). After sacrifice at 40 weeks, the number and size of tumors in each liver were quantified (Figure 5B). No difference was found in either tumor number or median tumor size between *Itgb8*^*flox/flox*^*;Alb-Cre* and control mice (Figure 5, C and D). This indicated that depletion of hepatocyte integrin αvβ8 did not predispose to increased tumor formation in this mouse model of HCC.

### Human Hepatocytes Express Integrin αvβ8 and Represent a Viable Therapeutic Target to Promote Liver Regeneration in Patients with Liver Disease

To assess the potential utility of integrin αvβ8 as a therapeutic target to promote hepatocyte proliferation and liver regeneration in patients with liver disease, the expression of integrin αvβ8 was assessed in samples of human liver. Uninjured liver tissue and tissue obtained from patients with acute liver failure secondary to acetaminophen overdose or from patients with cirrhosis were stained for the β8 integrin subunit. Widespread expression in hepatocytes was detected in all samples, demonstrating that integrin αvβ8 could be a viable potential therapeutic target in patients with a broad range of liver diseases (Figure 5E).

## Discussion

We show that depletion of hepatocyte integrin αvβ8 leads to increased hepatocyte proliferation and accelerated liver regeneration after partial hepatectomy in mice. The time course of hepatic *Itgb8* expression after partial hepatectomy, namely a rapid down-regulation followed by rebound up-regulation as the liver returns to its normal size, is consistent with a role for integrin αvβ8 as a brake on hepatocyte proliferation. This anti-proliferative role for integrin αvβ8 appears to be mediated via TGF-β, rather than altered hepatocyte adhesion, because blocking integrin αvβ8 on hepatocytes alters TGF-β–responsive gene expression. Of importance, the augmentation in hepatocyte proliferation in *Itgb8*^*flox/flox*^*;Alb-Cre* mice was not accompanied by increased susceptibility to hepatocellular tumor formation. Finally, human hepatocytes also express integrin αvβ8 in both acute and chronic liver disease; therefore, integrin αvβ8 represents a viable therapeutic target to promote liver regeneration in patients with a broad range of liver diseases.

Integrin αvβ8 has previously been shown to have a key regulatory role in the activation of latent TGF-β.^25^, ^26^, ^27^, ^28^, ^29^, ^30^, ^31^ The inhibitory effect of active TGF-β on hepatocyte proliferation is well established, including evidence demonstrating tonic inhibition of hepatocyte proliferation in the uninjured liver.^5^, ^6^, ^7^ The rapid down-regulation of hepatic *Itgb8* expression observed after partial hepatectomy is in line with the hypothesis that a reduction in integrin αvβ8-mediated activation of TGF-β is permissive for a pro-regenerative environment in the liver. To demonstrate subtle changes in activation status of TGF-β within the hepatic regenerative niche is challenging, given the magnitude and localized nature of these changes and also the small amount of remnant tissue present after two-thirds partial hepatectomy. However, inhibition of hepatocyte integrin αvβ8 *in vitro*, using a β8 integrin subunit blocking antibody, resulted in changes in expression of multiple TGF-β–responsive genes such as *Plat* and *Hmox1*. tPA, encoded by *Plat*, can activate HGF^51^ and has been shown to play a role in liver lobule reorganization after acute injury.^52^ Knockout of tPA in mice worsens injury after bile duct ligation, but this phenotype is reversed by administration of HGF.^53^ These data suggest that the regulatory role of integrin αvβ8 during hepatocyte proliferation is, at least in part, mediated via TGF-β signaling and that integrin αvβ8 depletion or inhibition may drive hepatocyte proliferation through tPA-mediated activation of HGF. Inhibition of hepatocyte integrin αvβ8 did not alter the proliferative response to stimulation with EGF and HGF *in vitro*, suggesting that the accelerated liver regeneration observed after hepatocyte integrin αvβ8 depletion does not occur via modulation of HGF or EGF downstream signaling pathways.

Detectable changes in the expression of genes that regulate the cell cycle were identified after partial hepatectomy, similar to those previously reported,^54^ but did not differ between *Itgb8*^*flox/flox*^*;Alb-Cre* mice and control mice. Inhibiting integrin αvβ8 *in vitro* also had no effect on expression of cell cycle genes, as measured by qPCR gene array. This would suggest that depletion of integrin αvβ8 on hepatocytes does not appear to change the kinetics of cell cycle regulation in the individual cell. Instead, depletion of hepatocyte integrin αvβ8 may promote liver regeneration by permitting a greater number of hepatocytes to escape the antiproliferative effects of active TGF-β. The observation of a reduction in the proportion of hepatic mitoses in *Itgb8*^*flox/flox*^*;Alb-Cre* mice compared with control mice at 72 hours after partial hepatectomy is difficult to reconcile with the increase in BrdU incorporation and the accelerated restoration of total liver mass. Although no change was detected in cell cycle kinetics, if the overall time in M phase were reduced in hepatocytes of *Itgb8*^*flox/flox*^*;Alb-Cre* mice, this could decrease the proportion of mitotic hepatocytes at any single point in time. Previous work has demonstrated a strong effect of circadian rhythm on hepatocyte entry into M phase after partial hepatectomy in mice, an effect not seen when assessing BrdU incorporation.^55^ However, in this study, partial hepatectomy was always performed in the morning and experimental order was randomized, so this should not account for differences between *Itgb8*^*flox/flox*^*;Alb-Cre* mice and control mice.

To screen for additional pathways that might be regulated by integrin αvβ8, gene expression was assessed by using microarray in whole liver samples from *Itgb8*^*flox/flox*^*;Alb-Cre* and control mice, comparing gene expression in uninjured liver and 24 hours after partial hepatectomy. Despite observing a large number of changes in gene expression (330) after partial hepatectomy that were restricted to *Itgb8*^*flox/flox*^*;Alb-Cre* mice, there were again no differences in expression of genes that regulated the cell cycle or proliferation compared with control mice. The failure to detect changes in expression of genes that regulated the cell cycle may also reflect limitations in sensitivity, particularly of the microarray technique, when applied to whole liver lysates from *Itgb8*^*flox/flox*^*;Alb-Cre* and control mice. Even after partial hepatectomy, only a minority of hepatocytes will be proliferating at any one time, and the presence of nonparenchymal cell mRNA in the whole liver lysates that were analyzed will further reduce the signal-to-noise ratio.

GO analysis of genes up-regulated only in *Itgb8*^*flox/flox*^*;Alb-Cre* mice after partial hepatectomy returned multiple terms that related to cytoskeletal organization and extracellular adhesion. Integrins are well known for their role in extracellular adhesion and their cytoplasmic domains can bind the cytoskeleton.^14^ However, it has previously been suggested that the cytoplasmic domain of the β8 subunit does not bind the cytoskeleton.^48^ Furthermore, no difference was found in the ability of hepatocytes isolated from *Itgb8*^*flox/flox*^*;Alb-Cre* and control mice to adhere to multiple extracellular matrix proteins found in both normal and regenerating liver.

Targeting of TGF-β pathways has been a major focus of research across several fields, particularly in the context of inflammation, wound healing, and oncogenesis. Unfortunately, global inhibition of TGF-β signaling can be associated with serious, undesirable effects, including excessive inflammation and development of neoplasia.^10^, ^11^, ^12^ This is highly likely to be due to the pleiotropic, context-dependent functions of TGF-β. Selective targeting of TGF-β activation by inhibition of integrin αvβ8 in the hepatic regenerative niche may potentially avoid many of the adverse effects noted with pan–TGF-β blockade while still promoting the desired effects on hepatocyte proliferation and liver regeneration. Of importance, these results did not demonstrate an increase in either hepatic inflammation or carcinogenesis in mice after depletion of hepatocyte integrin αvβ8.

Human hepatocytes express integrin αvβ8 in uninjured liver after acute hepatic injury secondary to acetaminophen overdose and also in cirrhosis. Therefore, hepatocyte integrin αvβ8 appears to be a viable translational target. There are potentially multiple clinical scenarios to which integrin αvβ8 inhibition could be applied. For example, using αvβ8 inhibition as a pro-regenerative therapy in the setting of acute liver failure may obviate the requirement for, or buy more time before, liver transplantation. Furthermore, combination with antifibrotic therapies could permit the restoration of functional, parenchymal liver mass in tandem with a reduction in fibrosis in patients with chronic liver disease. It might also allow more patients with primary or metastatic liver cancer to be treated successfully.

## Conclusion

Depletion of integrin αvβ8 on murine hepatocytes leads to increased hepatocyte proliferation and accelerated liver regeneration. Targeting integrin αvβ8 may therefore represent a promising therapeutic strategy to drive liver regeneration in patients with a broad range of liver diseases.

## Supplemental Data

Supplemental Table S1

Data Profile
