# Supplementary material for: Loss of Integrin αvβ8 in Murine Hepatocytes Accelerates Liver Regeneration
Source: Am J Pathol. 2019 Feb;189(2):258–71. doi: 10.1016/j.ajpath.2018.10.007 (PMC6360354; doi:10.1016/j.ajpath.2018.10.007)
Supplement: Supplemental Table S1 [file mmc1.docx]

**Supplementary Table S1. TGFβ signaling pathway array design.**

| **Gene Symbol** | **Name** | **Gene ID** |
| --- | --- | --- |
| *Actb* | actin, beta | 11461 |
| *Acvr1* | activin A receptor, type 1 | 11477 |
| *Acvr1b* | activin A receptor, type 1B | 11479 |
| *Afp* | alpha fetoprotein | 11576 |
| *Aldh1a1* | aldehyde dehydrogenase family 1, subfamily A1 | 11668 |
| *Aldob* | aldolase B, fructose-bisphosphate | 230163 |
| *Arhgap6* | Rho GTPase-activating protein 6 | 11856 |
| *Bcl2l14* | BCL2-like 14 (apoptosis facilitator) | 66813 |
| *Ccdc166* | coiled-coil domain containing 166 | 223648 |
| *Ccdc85a* | coiled-coil domain containing 85A | 216613 |
| *Ccna2* | cyclin A2 | 12428 |
| *Ccnd1* | cyclin D1 | 12443 |
| *Ccne1* | cyclin E1 | 12447 |
| *Cdkn1a* | cyclin-dependent kinase inhibitor 1a (p21) | 12575 |
| *Cdkn1b* | cyclin dependent kinase inhibitor 1b (p27) | 12576 |
| *Cdkn2b* | cyclin-dependent kinase inhibitor 2B (p15, inhibits CDK4) | 12579 |
| *Crct1* | cysteine-rich C-terminal 1 | 74175 |
| *Crebbp* | CREB binding protein | 12914 |
| *Ctgf* | connective tissue growth factor | 14219 |
| *Cxadr* | coxsackievirus and adenovirus receptor | 13052 |
| *Cyp2c70* | cytochrome P450, family 2, subfamily c, polypeptide 70 | 226105 |
| *E2f4* | E2F transcription factor 4 | 104394 |
| *Fn1* | fibronectin 1 | 14268 |
| *Furin* | furin (paired basic amino acid cleaving enzyme) | 18550 |
| *Gadd45b* | growth arrest and DNA-damage-inducible 45 beta | 17873 |
| *Gapdh* | glyceraldehyde-3-phosphate dehydrogenase | 14433 |
| *Gins2* | GINS complex subunit 2 (Psf2 homolog) | 272551 |
| *Glul* | glutamate-ammonia ligase (glutamine synthetase) | 14645 |
| *Gng13* | guanine nucleotide binding protein (G protein), gamma 13 | 64337 |
| *Gpd1* | glycerol-3-phosphate dehydrogenase 1 (soluble) | 14555 |
| *Gsta1* | glutathione S-transferase, alpha 1 (Ya) | 14857 |
| *Hcn2* | hyperpolarization-activated, cyclic nucleotide-gated K+ 2 | 15166 |
| *Hcrt* | hypocretin | 15171 |
| *Hist1h2bn* | histone cluster 1, H2bn | 319187 |
| *Hmgxb4* | HMG box domain containing 4 | 70823 |
| *Hmox1* | heme oxygenase 1 | 15368 |
| *Id2* | inhibitor of DNA binding 2 | 15902 |
| *Igfbp3* | insulin-like growth factor binding protein 3 | 16009 |
| *Igfbp4* | insulin-like growth factor binding protein 4 | 16010 |
| *Insc* | inscuteable homolog (Drosophila) | 233752 |
| *Itgav* | integrin alpha V | 16410 |
| *Itgb8* | integrin beta 8 | 320910 |
| *Izumo1r* | IZUMO1 receptor, JUNO | 64931 |
| *Jag1* | jagged 1 | 16449 |
| *Klf10* | Kruppel-Like Factor 10 | 21847 |
| *Ldlr* | low density lipoprotein receptor | 16835 |
| *Lgmn* | legumain | 19141 |
| *Maml2* | mastermind like 2 (Drosophila) | 270118 |
| *Mapk14* | mitogen-activated protein kinase 14 (p38a) | 26416 |
| *Mmp14* | matrix metallopeptidase 14 (membrane-inserted) | 17387 |

| **Gene Symbol** | **Name** | **Gene ID** |
| --- | --- | --- |
| *Mok* | MOK protein kinase | 26448 |
| *Mospd3* | motile sperm domain containing 3 | 68929 |
| *Myc* | myelocytomatosis oncogene | 17869 |
| *Net1* | neuroepithelial cell transforming gene 1 | 56349 |
| *Nfkbia* | nuclear factor of kappa light polypeptide gene enhancer in B cells inhibitor, alpha | 18035 |
| *Pdgfa* | platelet derived growth factor, alpha | 18590 |
| *Pdlim7* | PDZ and LIM domain 7 | 67399 |
| *Pigh* | phosphatidylinositol glycan anchor biosynthesis, class H | 110417 |
| *Plat* | plasminogen activator, tissue | 18791 |
| *Prg4* | proteoglycan 4 (megakaryocyte stimulating factor, articular superficial zone protein) | 96875 |
| *Psat1* | phosphoserine aminotransferase 1 | 107272 |
| *Rhob* | ras homolog family member B | 11852 |
| *Selenbp1* | selenium binding protein 1 | 20341 |
| *Sepp1* | selenoprotein P | 20363 |
| *Serinc2* | serine incorporator 2 | 230779 |
| *Serpine1* | serine (or cysteine) peptidase inhibitor, clade E, member 1 (PAI1) | 18787 |
| *Skil* | SKI-like | 20482 |
| *Slc26a1* | solute carrier family 26 (sulfate transporter), member 1 | 231583 |
| *Smad2* | SMAD family member 2 | 17126 |
| *Smad3* | SMAD family member 3 | 17127 |
| *Smad4* | SMAD family member 4 | 17128 |
| *Smad7* | SMAD family member 7 | 17131 |
| *Smurf2* | SMAD specific E3 ubiquitin protein ligase 2 | 66313 |
| *Sox4* | SRY (sex determining region Y)-box 4 | 20677 |
| *Sp1* | trans-acting transcription factor 1 | 20683 |
| *Sprr1a* | small proline-rich protein 1A | 20753 |
| *Sprr3* | small proline-rich protein 3 | 20766 |
| *Spryd4* | SPRY domain containing 4 | 66701 |
| *Stx5a* | syntaxin 5A | 56389 |
| *Sult1e1* | sulfotransferase family 1E, member 1 | 20860 |
| *Tfpi2* | tissue factor pathway inhibitor 2 | 21789 |
| *Tgfb1* | transforming growth factor, beta 1 | 21803 |
| *Tgfb3* | transforming growth factor, beta 3 | 21809 |
| *Tgfbr1* | transforming growth factor, beta receptor I | 21812 |
| *Tgfbr2* | transforming growth factor, beta receptor II | 21813 |
| *Tm7sf2* | transmembrane 7 superfamily member 2 | 73166 |
| *Tmsb4x* | thymosin, beta 4, X chromosome | 19241 |
| *Ugt1a9* | UDP glucuronosyltransferase 1 family, polypeptide A9 | 394434 |
| *Unc5b* | unc-5 netrin receptor B | 107449 |
| N/A | Genomic DNA control | N/A |
| N/A | Reverse Transcription control | N/A |
| N/A | Positive PCR control | N/A |
